# Extraosseous Accumulation of Technetium-99m-Methyl Diphosphonate (^99m^Tc-MDP) in a Child with ALL: A Case Report

**DOI:** 10.22038/aojnmb.2017.9679

**Published:** 2018

**Authors:** Farnaz Banezhad, Narjess Ayati, Farrokh Seilanian Toosi, Samine Boloursaz, S. Rasoul Zakavi

**Affiliations:** 1 Nuclear Medicine Research Centre, Mashhad University of Medical Sciences, Mashhad, Iran; 2 Department of Radiology, Mashhad University of Medical Sciences, Mashhad, Iran

**Keywords:** Acute lymphoblastic leukemia, Bone scan, Hypercalcemia, Lung uptake, Technetium-99m-methyl diphosphonate (^99m^Tc-MDP)

## Abstract

Extraosseous accumulation of technetium-99m-methyl diphosphonate (^99m^Tc-MDP) on bone scan is not common. This phenomenon is often attributed to abnormality of calcium metabolism and has been reported in a variety of conditions including metabolic diseases and malignancies. A five years old boy is presented here, who was admitted to the pediatric emergency suffering from fatigue, respiratory symptoms, weight loss, intermittent fevers, anorexia, nausea and vomiting, edema of legs and abdominal distension for one month. The initial laboratory analysis revealed hypercalcemia. The patient was referred for whole body bone scan with suspicion of malignancy and bone metastasis. The bone scan revealed highly increased radiotracer uptake in both lungs in the perfusion and blood pool phases. Delayed images also showed increased activity in lungs and gastric wall. The skeleton was not seen clearly. Bone marrow aspiration was done and established the diagnosis of ALL. The patient deceased due to respiratory failure 20 days later.

Diffuse lung uptake in this patient was consistent with respiratory failure and poor prognosis. It is reported that bone scan may be useful for assessment of the extent of metastatic calcification and may establish suitable management to prevent organ failure.

## Introduction

Extraosseous accumulation of technetium-99m-methyl diphosphonate (^99m^Tc-MDP) on bone scan is infrequent. This phenomenon is often attributed to abnormality of calcium metabolism and has been reported in a variety of conditions including metabolic diseases and malignancies (1). We are reporting a case of pediatric acute lymphoblastic leukemia (ALL) with highly increased ^99m^Tc-MDP uptake throughout both lungs.

## Case report

A five year old boy was admitted to the pediatric emergency department in a University Hospital, on 20^th^ September 2016, in a poor health condition with severe respiratory distress and severe weakness. The patient was a known case of failure to thrive (FTT) and had been suffering from fatigue, respiratory symptoms, weight loss, intermittent fevers, anorexia, nausea and vomiting, edema of legs and abdominal distension from one month earlier.

Clinical examination revealed an undernourished child with marked pallor, tachypnea, hepatomegaly and submandibular lymphadenopathy. The initial laboratory data is seen in table 1.

**Table 1:** Initial laboratory data of the child 7 days before bone scintigraphy

Parameter	Result	Reference Range	Parameter	Result	Reference Range
Calcium (mg/dl)	15.3	8.5-10.5	BUN (mg/dl)	63	5-23
Phosphate (mg/dl)	3.3	4-7	Cr (mg/dl)	0.7	4-7
Hemoglobin (g/dl)	6.9	12-15	AST (IU/L)	29	5-40
Leukocytes (10^9/L)	9	4-11	ALT (IU/L)	13	5-40
Platelets (10^9/L)	34	150-450	Total Protein (mg/dl)	6.9	6-8.5
ALP (IU/L)	759	180-200	Albumin (mg/dl)	3.8	3.5-5.5
LDH (IU/L)	1489	100-500	ESR (mm)	81	<15
PTH (pg/ml)	<2	14-72			

Packed cells and platelets were infused to compensate anemia and thrombocytopenia. Microbial cultures from blood, urine and cerebrospinal flood (CSF) samples were all negative for any bacterial growths.

In the initial CXR, consolidation was seen in the right lung. Otherwise cardiac silhouette was within the normal limits and musculoskeletal structures had no pathologic findings.

**Figure 1:**
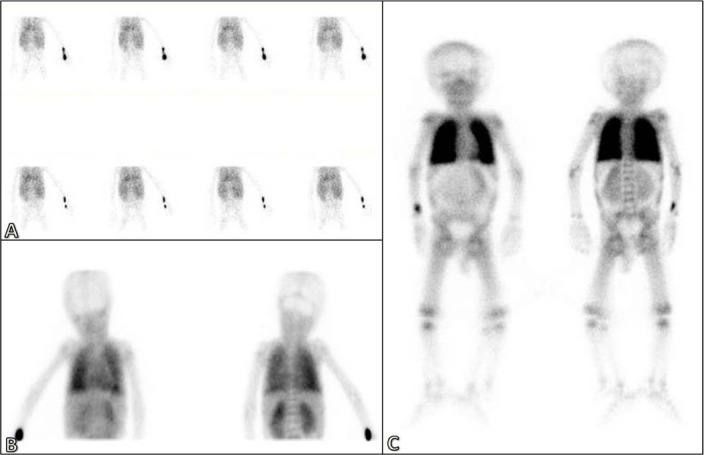
Perfusion (A) and blood pool (B) images showed increased tracer uptake in both lungs. Delayed images (C) also showed increased activity in the lungs and poor uptake in the skeleton

The patient was referred for whole body bone scan with suspicion of malignancy and bone metastasis. Immediately after intravenous injection of 74 MBq of ^99m^Tc-MDP, scanning was performed in 2 second intervals for 2 minutes from thoracolumbar region. Whole body blood pool images were also obtained immediately after early flow images for 5 minutes. Perfusion images were recorded in a matrix of 128×128. Multiple 750 Kilo counts spot views were recorded 2 and 3 hours later in a matrix of 256×256. All of these acquisitions were done with a dual-head E-CAM SPECT camera equipped with low energy, high resolution collimators and the SPECT images were performed with64 projections over 360°, 25 s/step, 64×64 matrix and noncircular orbit. The bone scan revealed highly increased activity in both lungs in the perfusion and blood pool phases (Figure 1. A, B). Delayed images also showed highly increased activity in lung and gastric wall and the skeleton was not seen clearly (Figure 1. C). This pattern suggested secondary calcification in the lungs and stomach. Considering hematologic findings in laboratory tests, hypercalcemia from a hematologic malignancy was the most probable diagnosis. Bone marrow aspiration was done and established the diagnosis of ALL. Lymphoblasts did not have myeloid markers and were positive for CD5 and CD7 which confirmed the diagnosis of T cell type of ALL.

Follow up blood examination showed elevated WBC count associated with presence of myelocytes, promyelocytes and band cells in the blood.

Abdominal ultrasonography revealed hepato-megaly. Both kidneys showed increased cortical echogenicity and absence of corticomedullary junction suggesting parenchymal renal disease.

**Figure 2:**
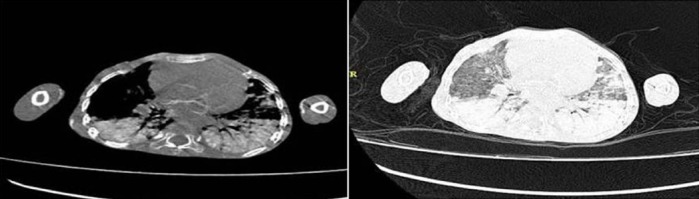
Chest CT showed bilateral hyperdense centrilobular nodules with ill-defined margin suggestive of metastatic calcification throughout both lungs

Chest CT showed bilateral hyperdense centrilobular nodules with ill-defined margin suggestive of metastatic calcification throughout both lungs (Figure 2). Differential diagnosis includes infectious/inflammatory process, malignancies (myeloproliferative disorders) and alveolar hemorrhage. CT bone window revealed lytic lesions involving body and posterior elements throughout the spine. Differential diagnoses for widespread lytic bone lesions were: metastasis, lymphoproliferative disorders, hyperparathyroidism (brown tumors), and infectious/inflammatory process.

Intravenous antibiotic therapy with Meropenem, Vancomycin and Co-Trimoxazole started with suspicion of pneumonia. Also treatment with furosemide and corticosteroid were done. No improvement in respiratory symptoms was noted and the patient passed away from respiratory insufficiency on Oct 11 2016. No autopsy was performed.

## Discussion

Whole body bone scan has been shown to be useful for detection of skeletal metastases in a variety of malignancies including ALL (1, 2). Diffuse lung uptake of ^99m^Tc-MDP has been reported in various diseases including parathyroid adenoma and carcinoma, multiple myeloma, vitamin D intoxication, carcinoma of bladder, T-cell leukemia, acute lymphoblastic leukemia, melanoma, acute and chronic renal failure, Wegener’s vasculitis, primary amyloid, Pneumocystis carinii pneumonia, Hodgkin’s lymphoma, Ewing’s sarcoma, Milk-Alkali syndrome and Waldenstrom’s macroglobulinemia (1, 3, 4).

Also, accumulation of ^99m^Tc-MDP in the stomach was documented in patients with multiple myeloma, vitamin D intoxication, acute lymphoblastic leukemia, melanoma, and acute and chronic renal failure (1).

Metastatic calcification is accumulation of calcium compounds in viable tissue, when hypercalcemia exists. A single mechanism is not responsible for soft tissue uptake of ^99m^Tc-MDP in different diseases and multiple factors may play a role. If there is deficiency in serum protein concentration and hypercalcemia is severe, precipitation of Ca compounds in extracellular space may occur. Also, alteration in PH is claimed to be responsible in calcification of other organs such as heart, liver, stomach and kidney (2).

The most common sort of leukemia among children is ALL which accounts for 80-85% of cases (5, 6). Signs and symptoms of ALL are nonspecific, such as fever, anemia, bleeding, hepatosplenomegaly, lymphadenopathy and bone and joint pain. Also CNS involvement, elevation of creatinine and uric acid and disruption of calcium and phosphorus metabolism are seen (4, 5). Hypercalcemia is a life-threatening disorder and a medical emergency that increase ALL morbidity significantly and if untreated may lead to cardiac arrhythmias, severe hypertension, renal failure, acidosis, dehydration and coma (4). Unfortunately the symptoms of hypercalcemia are nonspecific such as nausea, vomiting, anorexia, weight loss and fatigue, and may be mistaken by the symptoms of the main disease.

It has been reported that symptoms associated with hypercalcemia rather than hematologic abnormalities might lead to the diagnosis of leukemia (7).

Hypercalcemia occur in many childhood cancers such as rhabdomyosarcoma, hepatoblastoma, Hodgkin and non-Hodgkin’s lymphoma, brain tumors, neuroblastoma, angiosarcoma, acute lymphoblastic and myeloid leukemia (8). In adult patients, breast and lung cancers are the most common cancers in which hypercalcemia occurs, and myeloma is the most frequent among hematologic malignancies (9). Hypercalcemia is rarely seen in pediatrics and it accounts for 0.4%-1.3% of pediatric malignancies, while it accounts for 20%-30% of adult malignancies (4, 7).

To the extent of our knowledge, only two patients with ALL have been reported with extraosseous accumulation of ^99m^Tc-MDP. One of them was a 17-years-old boy with complaint of bone pain and hypercalcemia. Further evaluation with whole body bone scan using ^99m^Tc-MDP showed abnormal uptake in the spleen, both lungs and the kidneys (1). Another patient was an 18-years-old female presented with bone pain, abnormal weight bearing and hypercalcemia in whom bone scintigraphy showed abnormal tracer activity in the lungs, liver and kidneys with decreased tracer uptake throughout the skeleton (2). We did not find any report of a child of younger than 17 years old with ALL and high ^99m^Tc-MDP lung uptake in the literature.

This report demonstrates a rare case of ALL with hypercalcemia that causes soft tissue uptake of ^99m^Tc-MDP in the lungs and stomach. Highly increased diffuse lung uptake in this patient was consistent with respiratory failure and poor prognosis. It is reported that bone scan may be useful for assessment of the extent of metastatic calcification and may establish suitable management to prevent organ failure.

In conclusion, diffuse lung uptake of ^99m^Tc-MDP in pediatric patients with ALL may have important prognostic value.
